# Psychological Disorder and Patient Satisfaction in Aesthetic Surgery—A Systematic Review

**DOI:** 10.3390/medicina62020389

**Published:** 2026-02-16

**Authors:** Lavinia Hogea, Brenda Bernad, Amalia Marinca, Leonardo Corsaro, Iuliana Costea, Nina Ivanovic, Teodora Anghel

**Affiliations:** 1 Neuroscience Department, “Victor Babes” University of Medicine and Pharmacy, 300041 Timisoara, Romania; hogea.lavinia@umft.ro (L.H.); anghel.teodora@umft.ro (T.A.); 2 Neuropsychology and Behavioral Medicine Center, “Victor Babes” University of Medicine and Pharmacy, 300041 Timisoara, Romania; 3 Center for Studies and Research in Psychology, Faculty of Psychology, “Tibiscus” University, Lascăr Catargiu 4-6, 300559 Timisoara, Romania; 4 Department of Plastic Surgery, Università Campus Bio-Medico di Roma, Via Álvaro del Portillo, 21, 00128 Rome, Italy; leo_corsaro@hotmail.it; 5 Psychology Department, West University of Timisoara, 300223 Timisoara, Romania; iuliana.costea@e-uvt.ro; 6 Dermatology Department, “Victor Babes” University of Medicine and Pharmacy, 300041 Timisoara, Romania; nina.ivanovic@umft.ro; 7 Doctoral School of Medicine, “Victor Babes” University of Medicine and Pharmacy, 300041 Timisoara, Romania

**Keywords:** aesthetic surgery, psychological disorders, body dysmorphic disorder, cosmetic surgery patients, preoperative psychological assessment

## Abstract

*Background and Objectives*: This systematic review investigates the relationship between preoperative psychological disorders and postoperative satisfaction among patients undergoing aesthetic surgery. While aesthetic procedures can enhance self-image, growing evidence indicates that underlying mental health conditions, particularly BDD, depression, and anxiety, may compromise surgical outcomes. *Materials and Methods*: A comprehensive literature search was performed in PubMed, the Cochrane Library, and Google Scholar between January 2010 and December 2024. Eligible observational studies assessed preoperative psychological conditions—primarily body dysmorphic disorder, depression, and anxiety—using validated instruments, such as the Body Dysmorphic Disorder Questionnaire (BDDQ), Body Dysmorphic Disorder Examination (BDDE), Hospital Anxiety and Depression Scale (HADS), Beck Depression Inventory (BDI), and structured clinical interviews, and reported postoperative patient-reported satisfaction following aesthetic surgery. Study quality was evaluated using an adapted QUIPS framework. *Results*: Across the 13 included studies, six reported a negative association between moderate-to-severe preoperative psychological symptoms and postoperative satisfaction, five found no significant association, and two described positive or conditional associations. Methodological heterogeneity in psychological assessment tools and satisfaction measures was a major source of divergence across studies. Despite these differences, the evidence underscores the need for standardized, validated psychological evaluation protocols in aesthetic surgery. Incorporating mental health screening into routine surgical planning can enhance ethical practice, reduce dissatisfaction, and improve long-term patient outcomes. *Conclusions*: These findings advocate for a multidisciplinary approach that includes psychological assessment as an essential component of patient care in aesthetic medicine.

## 1. Introduction

In plastic surgery, aesthetic procedures are typically understood as interventions aimed at enhancing physical appearance. In contrast, plastic surgery more broadly includes both reconstructive and aesthetic components that seek to restore function and appearance following trauma, illness, or congenital anomalies. Cosmetic surgery is invasive in enhancing a patient’s perceived physical attractiveness [1]. Research has demonstrated that in some demographic groups, for example, in Saudi Arabia, a growing interest in cosmetic surgery correlates with a higher prevalence of body dysmorphic disorder (BDD) [2].

BDD is a mental health condition in which an individual is excessively preoccupied with one or more perceived flaws in their physical appearance that are often barely noticeable or non-existent to others. BDD is classified within the obsessive-compulsive and related disorders in the Diagnostic and Statistical Manual of Mental Disorders, Fifth Edition (DSM-5) [3,4]. The essence of BDD centers on a persistently negative body image, which can provoke a multitude of challenges, including severe anxiety, depression, and social isolation [5]. The prevalence of BDD in the general population is estimated at 1–2%. At the same time, it is markedly higher in populations seeking cosmetic procedures, with prevalence rates reportedly reaching 13.2% in cosmetic surgery clinics [6].

Cultural contexts help define beauty ideals and influence how individuals modify their appearance [7]. This dynamic is particularly well-illustrated by Rhee et al., who found that many Asian individuals undergo cosmetic surgery to attain social mobility or a professional edge, revealing how culturally driven beauty standards fuel demand for aesthetic procedures [8].

Aesthetic surgery has become a means for individuals, particularly in aging populations, to maintain a youthful image, reflecting a broader connection between societal ideals of beauty and concepts of wellness [9]. The psychological outcomes of cosmetic surgery are complex. While some studies report positive effects on self-image and emotional well-being [10,11], others highlight risks such as persistent dissatisfaction and psychological distress, revealing the nuanced relationship between aesthetic procedures and mental health [12,13].

The frequent occurrence of psychological conditions such as anxiety and depression in individuals considering cosmetic procedures calls for a multidisciplinary approach in clinical practice [11]. Mental health professionals and plastic surgeons should work collaboratively, particularly given that Mendes et al. have identified a significantly higher rate of psychological disorders among aesthetic surgery candidates compared to the general population [14].

Although a growing body of literature has explored the association between aesthetic surgery and psychological disorders, existing evidence remains heterogeneous and methodologically fragmented. Prior studies vary substantially in how psychological conditions are defined and assessed, how postoperative satisfaction is measured, and whether preoperative screening protocols are applied systematically. Importantly, no consensus exists regarding standardized psychological assessment protocols or their integration into aesthetic surgical practice. This systematic review addresses these gaps by providing a structured synthesis of available evidence, explicitly mapping heterogeneous outcome measures to postoperative satisfaction and critically evaluating the adequacy and consistency of preoperative psychological screening across studies. In doing so, the review offers an original contribution by clarifying sources of inconsistency in the literature and identifying key methodological limitations that currently hinder evidence-based patient selection and clinical decision-making in aesthetic surgery.

For the purposes, the term “psychological disorder” refers to either (a) a formally diagnosed psychiatric condition established through structured clinical interviews based on recognized diagnostic criteria, or (b) clinically relevant psychological symptomatology identified preoperatively using validated screening instruments with established cut-off thresholds (e.g., BDDQ, HADS, BDI, BDDE). Studies relying solely on informal clinical impressions without validated assessment tools were not considered sufficient for inclusion. Given the heterogeneity in how postoperative satisfaction is assessed in aesthetic surgery research, outcomes were categorized a priori into conceptually related domains. Direct patient-reported satisfaction measures (e.g., global satisfaction scales, procedure-specific satisfaction questionnaires) were distinguished from indirect or proxy constructs, including health-related quality of life, body image disturbance, dysmorphic concern, and symptom-based self-reported distress. This approach was adopted to avoid overgeneralization and to ensure conceptual alignment with the primary research question.

Despite increasing interest in psychological factors in aesthetic surgery, existing studies report divergent findings and apply heterogeneous definitions of both psychological disorders and postoperative satisfaction, with no standardized approach to psychological screening or outcome mapping. This lack of methodological consistency limits the interpretability and clinical applicability of current evidence.

This review aims to evaluate the impact of preoperative psychological disorders on postoperative satisfaction among aesthetic surgery patients and to assess the adequacy of current psychological assessment practices used in this context. Specifically, the review seeks to (1) identify and classify the most frequently reported psychological conditions among cosmetic surgery candidates; (2) examine how these conditions influence postoperative satisfaction and perceived surgical outcomes; and (3) assess the quality and consistency of preoperative psychological screening methods used in aesthetic surgical practice. By addressing these objectives, this review aims to examine how preoperative psychological disorders affect postoperative satisfaction in aesthetic surgery and evaluate the adequacy of preoperative psychological screening practices.

## 2. Materials and Methods

### 2.1. Search Strategy

This systematic review used recognized methodological standards. To ensure transparency, the study protocol was prospectively registered (PROSPERO ID: CRD420251068477), and the reporting followed the PRISMA (Preferred Reporting Items for Systematic Reviews and Meta-Analyses) guidelines [15,16].

A systematic literature search was conducted using major databases: PubMed, Cochrane Library, and Google Scholar. The search strategy was developed to identify relevant studies on psychological disorders associated with aesthetic surgery. The electronic search strategy was developed in advance and adapted for each database. In PubMed, the following search string was used: (MeSH terms): (“cosmetic surgery” [MeSH] OR “aesthetic surgery” OR “plastic surgery”) AND (“psychological disorders” [MeSH] OR “body dysmorphic disorder” OR depression OR anxiety) AND (“patient satisfaction” OR “treatment outcome”). Filters were applied to include human studies, English-language publications, and articles published between January 2010 and December 2024. In the Cochrane Library, the search strategy used a comparable combination of keywords and subject headings, adapted to the database syntax: (“aesthetic surgery” OR “cosmetic surgery”) AND (“psychological disorder” OR “body dysmorphic disorder” OR depression OR anxiety) AND (“patient satisfaction”). Given the limited reproducibility and ranking-based retrieval of Google Scholar, a standardized screening approach was applied. Searches were conducted using the same keyword combinations as in PubMed, and results were sorted by relevance. Only the first 200 records retrieved were screened, in accordance with methodological recommendations for systematic reviews. Titles and abstracts were assessed independently by two reviewers, and records identified exclusively through Google Scholar were required to meet all predefined eligibility criteria to be included. All database searches were last conducted from 15 June 2025 to 15 October 2025. The search was restricted to studies published between January 2010 and December 2024 to ensure that the review captured the most relevant and updated research. Eligible articles included original research with observational designs such as prospective and retrospective cohort studies, cross-sectional studies, and case–control studies. Only peer-reviewed English-language articles involving human participants were considered. Only studies focusing on psychological disorders in aesthetic surgery patients were chosen.

### 2.2. Study Selection

**Figure 1:**
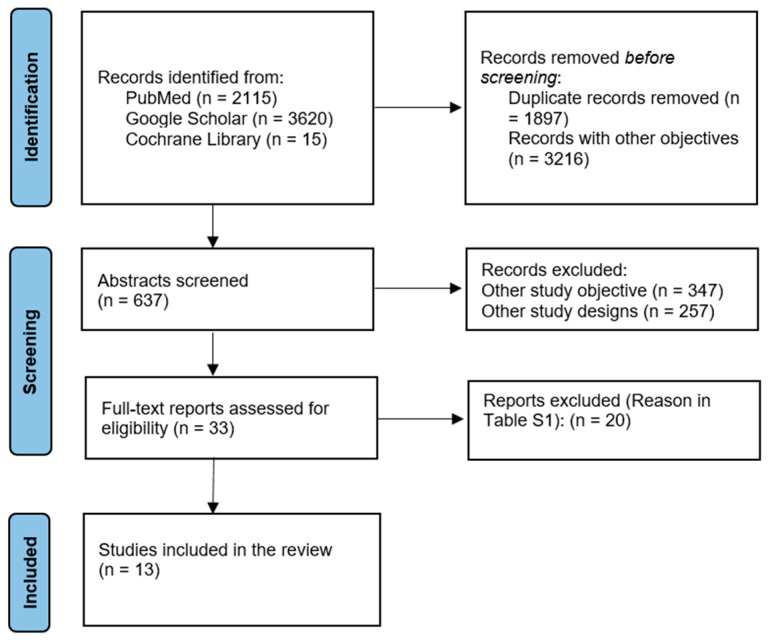
Study selection according to PRISMA guidelines.

Two researchers independently screened the titles, abstracts, and full-text articles to determine whether studies were eligible for inclusion in the review. The selection process is illustrated in Figure 1, following PRISMA guidelines.

The inclusion criteria for studies were as follows: (1) participants had to be 18 years of age or older; (2) the research needed to focus exclusively on elective aesthetic or cosmetic surgical procedures, excluding emergency surgeries, medically necessary operations, or non-surgical treatments; and (3) patients presenting with pre-existing psychological conditions identified preoperatively, either through a formal clinical diagnosis or through validated psychological screening instruments with defined diagnostic or severity thresholds (e.g., BDDQ, HADS, BDI, BDDE). (4) Only original research articles and peer-reviewed using primary study designs—such as prospective or retrospective cohort studies, cross-sectional studies, or case–control studies—were included. Additionally, (5) eligible studies had to measure and report patient satisfaction outcomes and involve a minimum sample size of 10 participants, (6) in English.

The exclusion criteria for studies were: (1) those addressing non-surgical cosmetic procedures or medically indicated surgeries and (2) studies lacking a psychological assessment conducted before surgery. (3) Articles that did not report on patient satisfaction or failed to provide precise methodological details about psychological evaluation or study design were also excluded from the review.

### 2.3. Data Analysis

Data extraction was performed independently by two human reviewers using a standardized extraction form. Elicit AI was used solely as a supportive tool to identify relevant text passages and did not replace human judgment. A predefined prompting protocol was applied consistently across studies to extract study characteristics, psychological assessment methods, and outcome measures. All extracted data were verified manually against the full-text articles. Discrepancies between reviewers were resolved through discussion and consensus. Given that the original study authors were not contacted for clarification, a full manual audit of all extracted variables was conducted prior to final synthesis. The assistant was programmed with detailed instructions to extract specific variables, including study design, psychological assessment methods, satisfaction measurement tools, participant demographics, and reported outcomes. Although the automation tool supported data structuring and consistency, human evaluators reviewed and verified the final data entries to ensure accuracy. No direct contact was made with the study authors for additional clarification or data verification.

The primary outcome of interest was patient satisfaction following aesthetic surgery. Data were sought on how satisfaction was measured, including the specific instruments used (e.g., Visual Analog Scale, BODY-Q, Rhinoplasty Outcome Evaluation), timing of the assessment (e.g., short- or long-term post-surgery), and scale details (e.g., scoring range, interpretation). Where available, all relevant satisfaction outcomes reported in each study, across different measurement tools and time points, were extracted. When multiple satisfaction metrics were presented, preference was given to validated, surgery-specific tools. Studies that lacked clear definitions or descriptions of satisfaction measures were noted accordingly.

Additional data items extracted included participant characteristics such as age, gender, sample size, and recruitment settings, as well as the psychological conditions diagnosed preoperatively, including BDD, depression, and anxiety. The data also covered the psychological assessment tools used across studies, including the Beck Depression Inventory, the Hospital Anxiety and Depression Scale (HADS), the Body Dysmorphic Disorder Questionnaire (BDDQ), and the Situational Inventory of Body Image Dysphoria (SIBID). Information regarding the study design and setting was also collected. Additionally, details about the primary aesthetic procedure types were recorded. When specific information was missing or unclear, no assumptions were made; such data points were marked as “N/A” to ensure clarity and transparency in reporting.

Preoperative psychological assessment varied across studies and included validated psychometric instruments, structured clinical interviews, and documented psychiatric diagnoses derived from medical records. Studies relying exclusively on informal impressions without documented preoperative psychological evaluation were excluded. Variability in assessment modality was retained to reflect real-world clinical practice and is addressed as methodological heterogeneity.

### 2.4. Risk of Bias Methodology

We evaluated the risk of bias in the included studies using the Quality In Prognosis Studies (QUIPS) tool [17], which was adapted to our review’s specific needs (see Supplemental Materials, Tables S2 and S3). This tool assesses six domains: study participation (D1), study attrition (D2), measurement of the prognostic factor (D3), outcome measurement (D4), control for confounding variables (D5), and statistical analysis and reporting (D6). Following the QUIPS framework, each domain in each study was classified as having a “low,” “moderate,” or “high” risk of bias. Risk-of-bias assessment was performed independently by two authors. Automated tools were used solely for initial data organization and did not contribute to final bias judgments. Discrepancies were resolved through consensus.

### 2.5. Statistical Analysis

The statistical analysis evaluated the relationship between pre-existing psychological conditions and postoperative satisfaction in aesthetic surgery patients. Data were extracted from studies that reported quantitative outcomes, including correlation coefficients, *p*-values, and validated psychometric measures. Where available, the strength and direction of associations between variables such as BDD, depression, or anxiety and patient-reported satisfaction were analyzed. Studies using standardized tools and validated scales were prioritized to ensure methodological consistency. The analysis incorporated both bivariate (Pearson or Spearman correlation) and multivariate findings, which were reported. Effect sizes and statistical significance levels (*p* < 0.05) were noted to determine the robustness of reported associations.

The analysis was performed using Python (version 3.11), utilizing structured outputs and natural language processing tools integrated within the Elicit AI platform.

## 3. Results

### 3.1. Overview Under Selected Studies

An initial search across electronic databases yielded 5750 records. After removing 1897 duplicate entries and 3216 records deemed irrelevant based on title and scope (e.g., studies not addressing psychological factors or satisfaction outcomes in aesthetic surgery), 637 abstracts remained for screening. Of these, 347 studies were excluded for addressing unrelated research objectives, while an additional 257 were removed due to incompatible study designs (e.g., reviews, editorials, or case reports). This left 33 full-text articles for detailed eligibility assessment. After a full-text review, 20 studies were excluded for insufficient data, unclear psychological assessment methods, or failure to report patient satisfaction outcomes (see Table S1 in the Supplemental Materials). Ultimately, 13 studies published between 2010 and 2024 met all inclusion criteria, with 2643 patients, and were included in the final systematic review [14,18,19,20,21,22,23,24,25,26,27,28,29,30] (Table 1).

The studies varied in design, with most being prospective or observational cohort studies, while several used cross-sectional methodologies. The number of patients included in the studies ranged from 31 to 544, with a median of 115 (25th–75th percentiles 84–204). The surgical procedures ranged from rhinoplasty and breast augmentation to various facial and body aesthetic interventions. Studies reflect diversity across countries in clinical and research settings (Germany, Brazil, Australia, the USA, Norway, China, and Italy).

Looking at procedure type, rhinoplasty was the most frequently studied procedure (5 studies) [18,19,20,21,29], followed by various cosmetic/aesthetic surgeries (4 studies) [14,23,24,30]. Other methods included facelifts [25,26], breast implants [27], and abdominoplasty [28].

Psychological assessments varied significantly across studies, with notable differences in the choice and application of instruments. The most frequently used tools included (in six studies) the BDD [18,19,20,26,28,29], the HADS [25], the BDI, and the RSE [25]. Additional instruments such as the SIBID [30], the BSQ [28], and the SF-36 [30] were employed to evaluate quality of life and body image perception. While some studies incorporated multiple validated scales alongside structured clinical interviews [14,21,26,27].

### 3.2. Risk of Bias

Across all included studies, risk of bias was assessed using QUIPS, and the analysis presented in Table S3 (Supplementary Material) offers critical insights.

Studies such as Hessler (2010), Von Soest (2011), Felix (2014), de Brito (2016), Mendes (2023), and Losorelli (2024) [14,19,20,23,26,28] demonstrated overall low risk of bias across nearly all domains. These studies had well-defined participant selection, reliable psychological assessments, consistent satisfaction measurement, and thorough statistical reporting. Their strength lies in the use of validated instruments (e.g., BDDQ, BDDE), clearly described recruitment processes, and robust outcome analysis. Such studies provide the most methodologically sound contributions to the synthesis of evidence.

Picavet (2013), Gabrielyan (2015), and Bresnick (2024) [18,27,30] showed moderate risk in domains related to measuring psychological variables and adjusting for confounders. While these studies reported some form of psychological evaluation and outcome tracking, they lacked comprehensive descriptions of their assessment tools or failed to sufficiently account for confounding variables such as age, procedure type, or pre-existing mental health severity. These limitations reduce the reliability of observed associations between psychological factors and satisfaction.

Honigman (2011), Constantian (2014), and Wei (2024) [21,25,29] emerged as the studies with the highest risk of bias, particularly in the domains of psychological assessment, confounding adjustment, and study attrition. For instance, Honigman (2011) and Constantian (2014) [21,25] used vague or inadequately reported psychological measures, and both failed to appropriately adjust for relevant confounders in their statistical models. Wei (2024) [29] was the only study with a high statistical analysis and reporting risk, indicating substantial concerns regarding selective reporting or insufficient transparency.

Across the studies, the most common sources of bias were study attrition (D2), due to incomplete follow-up reporting; inadequate adjustment for confounders (D5), with limited control of key variables; and inconsistent use of validated tools for psychological assessment (D3).

### 3.3. Relationship Between Psychological Conditions and Satisfaction

**Figure 2:**
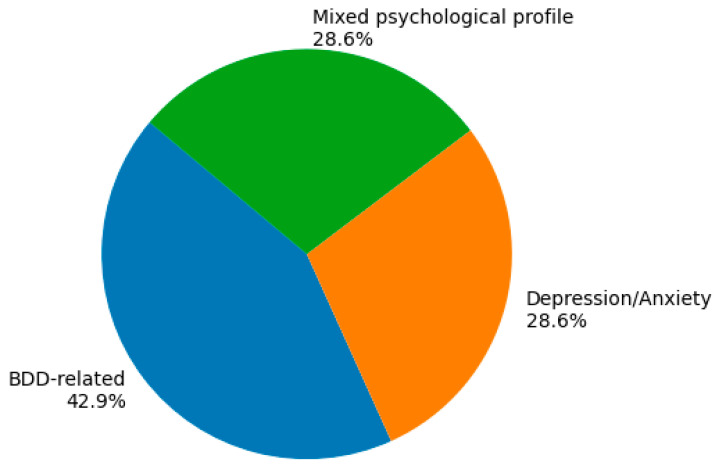
Psychological Condition Evaluated Across Studies.

The relationship between pre-existing psychological conditions and postoperative satisfaction was examined using a descriptive statistical synthesis, as substantial heterogeneity across study designs, psychological assessment instruments, and satisfaction outcome measures precluded quantitative meta-analysis. Consequently, studies were analyzed at the study level, with a focus on the psychological constructs assessed and the direction and statistical significance of their reported associations with postoperative satisfaction. As shown in Figure 2, body dysmorphic disorder (BDD)–related constructs were the most frequently evaluated psychological domain, being assessed in six studies [18,19,20,28,29,30]. Depression and anxiety were explicitly examined in four studies, while an additional four studies evaluated mixed or broader psychological profiles, including global mental health, body image dysphoria, or quality-of-life–related measures [21,25,26,27]. This distribution highlights the predominant emphasis of the literature on appearance-related psychopathology when investigating satisfaction outcomes following aesthetic surgery.

**Figure 3:**
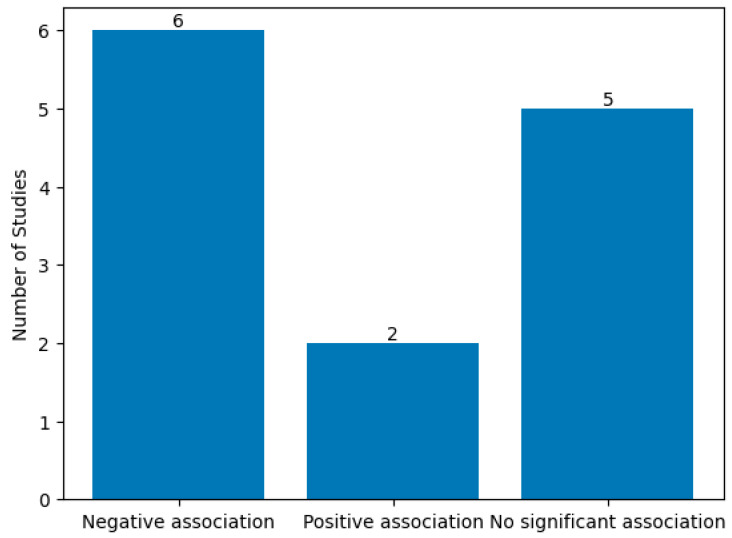
Reported relationship between psychological condition and satisfaction.

The direction of the reported association between psychological conditions and postoperative satisfaction is summarized in Figure 3. A statistically significant negative association was reported in six of the thirteen included studies (46.2%), indicating that higher psychological symptom burden was frequently associated with lower postoperative satisfaction. Specifically, Honigman et al. (2011) demonstrated inverse relationships between depression and anxiety scores and satisfaction outcomes [25], while von Soest et al. (2011) reported poorer satisfaction among patients with preoperative psychological problems [23]. Picavet et al. (2013) identified moderate negative correlations between BDD severity and satisfaction following rhinoplasty (ρ = −0.43 to −0.48, *p* < 0.001) [18], and Gabrielyan et al. (2015) reported a strong inverse association between body image dysphoria and mental health–related quality-of-life outcomes (r = −0.7, *p* < 0.05) [30]. Similarly, Constantian et al. (2014) observed reduced patient-defined surgical success among individuals with depressive symptoms [21], and Losorelli et al. (2024) found significantly lower aesthetic satisfaction in patients screening positive for BDD (*p* < 0.002) [20].

In contrast, a positive association between psychological status and postoperative satisfaction was identified in two studies (15.4%). Hessler et al. (2010) reported higher satisfaction among patients receiving treatment for depression (*p* = 0.05) [26], while Felix et al. (2014) observed high postoperative satisfaction in patients with mild to moderate BDD following rhinoplasty, although no direct effect size was reported linking depression or anxiety to satisfaction [19].

Five studies (38.4%) reported no statistically significant association between psychological conditions and postoperative satisfaction. These included Margraf et al. (2013), which found no relationship between psychological outcomes and goal attainment; de Brito et al. (2016), which reported no significant association between BDD symptoms and postoperative outcomes; Mendes et al. (2023), which focused on the prevalence of psychological disorders rather than satisfaction outcomes; Wei et al. (2024), in which indirect satisfaction proxies did not differ significantly by BDD screening status; and Bresnick et al. (2024), which primarily examined breast implant illness rather than satisfaction per se [14,24,27,28,29].

Only four studies (30.8%) provided quantifiable effect size estimates, including correlation coefficients or statistically significant *p*-values, while the remaining studies reported qualitative associations or null findings without effect size reporting. Overall, this descriptive synthesis indicates that nearly half of the included studies identified a significant negative association between psychological symptom severity and postoperative satisfaction, whereas positive associations were uncommon and largely confined to settings involving mild or treated psychological conditions. The substantial methodological heterogeneity and incomplete reporting of effect sizes across studies prevented statistical pooling and underscore the need for more standardized psychological and satisfaction outcome assessment in future research.

## 4. Discussion

### 4.1. Summary of Results

This review highlights that the relationship between psychological disorders and postoperative satisfaction is not uniform, but influenced by symptom severity, assessment rigor, and methodological design. An important methodological consideration is the conceptual heterogeneity of postoperative satisfaction across studies. While direct satisfaction measures provide the most straightforward alignment with the research question, proxy constructs such as body image disturbance or quality-of-life impairment capture broader dimensions of postoperative experience. These constructs should not be interpreted as interchangeable, and associations observed across studies should be understood as reflecting related but distinct aspects of patient-perceived outcomes.

These findings suggest that psychological vulnerability is not inherently incompatible with satisfactory outcomes but requires appropriate identification and management. It also highlights that not all psychological disorders predict dissatisfaction; context and severity matter [19,26].

Null findings across several studies are more plausibly explained by methodological limitations—such as inadequate psychological assessment, poor confounder control, or indirect outcome measures—than by the absence of a true association.

### 4.2. Correlation with Literature

Its suggest that individuals pursuing aesthetic procedures tend to show higher levels of psychological distress than the general population [31,32]. Symptoms of depression, anxiety, and particularly BDD are frequently present and often linked to unrealistic expectations of surgical results, and psychological distress significantly influences surgical decision-making, and those with depressive traits may be more prone to postoperative dissatisfaction [32,33].

Higher preoperative anxiety is also associated with lower postoperative satisfaction. Similar patterns have been observed in other surgical fields, where depression before surgery often predicts poorer satisfaction outcomes [34]. These findings underscore the importance of psychological screening and support during the preoperative process to improve the surgical experience [35].

The phenomenon of “surgery insatiability” points to deeper psychological issues when patients repeatedly seek procedures due to unresolved dissatisfaction, often tied to unmet emotional needs rather than physical outcomes [36]. This highlights the necessity for a comprehensive psychological evaluation to ensure realistic expectations. While some may experience temporary distress, many report long-term improvements in well-being when their psychological state is taken into account [37,38]. Ultimately, not all patients achieve psychological benefits post-surgery. Satisfaction is deeply influenced by individual expectations and their perception of improvement, making preoperative dialogue and mental health screening vital [39].

The included studies displayed notable methodological variability, particularly in design and psychological assessment approaches. While some employed validated tools such as the BDDQ, HADS, and BDI [20,25,26], others lacked formal evaluations or relied on unvalidated methods, weakening their reliability [21,27]. The timing of assessments also varied, with limited use of structured clinical interviews. Studies using validated tools generally reported clearer links between psychological disorders and satisfaction, while those with poor screening practices often found inconclusive results. This highlights the importance of standardized, high-quality psychological evaluation in aesthetic surgery research and practice.

Implementing standardized psychological screening protocols in aesthetic surgery will be a must to improve patient safety and clinical outcomes. Many individuals seeking cosmetic procedures present with psychological conditions, particularly BDD, which is notably prevalent in this population. Research suggests that up to 13.2% of cosmetic surgery patients may suffer from BDD, increasing the risk of poor outcomes if these individuals undergo surgery without proper evaluation [40,41]. Early psychological assessment helps identify at-risk patients and supports more responsible surgical decision-making [31,42]. Ethically, performing procedures on patients with untreated mental health issues, such as BDD or depression, raises concerns. Without screening, surgeries may worsen psychological distress rather than alleviate it [35,43]. Comprehensive assessments uphold ethical standards and center on patient welfare [31].

A multidisciplinary approach is recommended, using validated screening tools and involving mental health professionals. Frameworks like the SAGA mnemonic help clinicians understand a patient’s psychological readiness and expectations [44,45]. Aligning surgical goals with psychological resilience can reduce dissatisfaction [24,35]. Managing expectations is also key. Transparent communication, patient education, and strong social support systems encourage emotional preparedness and satisfaction [35,46]. Informed consent must reflect both physical outcomes and psychological implications [47]. Understanding the complexities of body image helps prevent poor outcomes and supports more ethical, patient-centered care [42,48,49,50].

Findings from this systematic review reveal both convergence and divergence in understanding the psychological factors that influence patient satisfaction in aesthetic surgery, as supported by the literature. This research systematically demonstrates that preoperative psychological conditions tend to correlate with lower postoperative satisfaction, especially when these conditions are moderate to severe and left unaddressed. This conclusion is strongly supported by the review by Honigman and colleagues, who emphasize that although many cosmetic surgery patients report satisfaction with aesthetic outcomes, this does not necessarily translate into improvements in psychological well-being [11]. Their analysis highlights that patients with a history of depression, anxiety, or unrealistic expectations are at elevated risk of poor psychosocial adjustment post-surgery. Importantly, the included studies primarily assessed patient-reported satisfaction outcomes and did not systematically report objective indicators of technical surgical success. Therefore, the observed associations reflect patients’ subjective perceptions of outcomes rather than independently verified surgical performance [11]. These findings align closely with the conclusions of this review, which stress the importance of rigorous psychological assessment before surgical intervention to help avoid such negative outcomes.

Similarly, Mulkens et al. (2012) provide quantitative data that reinforce these insights [51]. Their study shows that patients exhibiting higher symptoms of BDD report lower satisfaction with surgical results and present with elevated levels of general psychopathology and lower self-esteem [51]. This supports the idea articulated in this research that untreated or unrecognized psychological disorders may not only fail to improve postoperatively but can be exacerbated by the surgical process [51]. Their work also highlights the failure of current preoperative screening to identify such vulnerable individuals; a concern echoed in both Jafferany’s and Honigman’s reviews.

Jafferany et al. (2020) further elaborate on the high prevalence of psychiatric disorders among patients seeking aesthetic interventions [52]. According to their findings, nearly half of these patients may meet diagnostic criteria for a mental health disorder, most commonly BDD, narcissistic personality disorder, or histrionic personality disorder [52]. Their article underlines the necessity of psychological screening and early detection, particularly given that many individuals with these disorders are not aware of their psychological challenges and may seek repeated or unnecessary surgeries [52]. This further substantiates the central thesis of this study, which advocates structured, standardized psychological evaluations as an ethical and clinical imperative.

However, not all reviewed studies draw direct links between psychological disorders and dissatisfaction. For example, Dreher et al. (2016) focus primarily on quality-of-life (QoL) outcomes after aesthetic procedures [37]. Their meta-analysis shows a general improvement in QoL metrics across several types of cosmetic surgeries, with reduction mammoplasty appearing to yield the most consistent gains in both physical and social functioning domains [37]. Unlike this research, Dreher and colleagues do not focus on psychological disorders as predictive variables. Instead, their contribution suggests that surgical interventions tend to have beneficial outcomes for many patients in a general aesthetic population. While this does not contradict this study, it highlights that psychological distress may not be universal among cosmetic surgery seekers, and that the presence or absence of psychological comorbidity is key to predicting outcome variability.

Despite the different angles and methodologies, the combined evidence from all sources leads to a coherent conclusion: psychological factors play a significant role in shaping the patient’s experience and satisfaction with aesthetic surgery. When psychological conditions such as BDD, depression, or anxiety are present and unaddressed, they are likely to undermine the patient’s postoperative satisfaction, regardless of the objective QoL surgical outcome. Conversely, when patients are appropriately assessed and supported through a multidisciplinary approach, surgery can benefit their self-image and QoL.

### 4.3. Strengths and Limitations of the Study: Future Direction

This systematic review offers several important strengths that enhance its rigor, relevance, and potential impact. First, it follows a transparent and structured methodology, adhering to PRISMA guidelines and using a registered protocol. Including only peer-reviewed original studies with defined psychological assessments and reported satisfaction outcomes enhances methodological robustness. Another strength lies in the diversity of data sources, encompassing studies from various countries and surgical contexts, which supports the generalizability of findings. The use of validated psychological tools (such as the BDDQ, HADS, BDI, and SIBID) in many of the included studies adds credibility to the findings, while the application of the QUIPS tool for assessing risk of bias further contributes to a nuanced interpretation of the literature.

Moreover, the review synthesizes results from a broad range of psychological conditions (including BDD, depression, and anxiety). It directly links these to postoperative satisfaction, offering a more comprehensive perspective than many previous studies. Including both bivariate and multivariate findings, along with a structured analysis of effect sizes, strengthens the interpretability of correlations across diverse methodologies.

Nonetheless, several limitations must be acknowledged. The included studies displayed considerable heterogeneity in design, psychological screening methods, and satisfaction measurement tools. The absence of standardized, objective measures of surgical technical success across studies limits conclusions regarding the independence of psychological factors from procedural outcomes. This variability limited the feasibility of performing a formal meta-analysis or deriving pooled effect sizes. Additionally, the reliance on self-reported psychological diagnoses and the lack of structured clinical interviews introduced misclassification bias. Publication bias is also a concern, as studies reporting non-significant or negative findings may be underrepresented in the literature. Furthermore, some included studies had small sample sizes or poor reporting standards, which constrained the strength of conclusions that could be drawn from them. Finally, the cross-sectional design of several studies precludes definitive causal inferences regarding the relationship between psychological status and postoperative satisfaction. This review also highlights key gaps in the literature, offering a roadmap for future research to optimize patient selection, manage expectations, and ultimately improve surgical outcomes through integrative mental health evaluation.

Future research should prioritize longitudinal, multi-center studies that systematically assess the psychological profiles of aesthetic surgery candidates using validated diagnostic tools. Many current studies rely on self-report instruments or lack preoperative assessments, limiting the reliability and generalizability of their findings. Standardizing psychological evaluation protocols across clinical settings would enhance comparability between studies and provide more accurate estimates of how specific disorders impact postoperative satisfaction. There is also a need to explore the effectiveness of preoperative psychological interventions, such as counseling or cognitive-behavioral therapy, in improving surgical outcomes for high-risk patients. Investigating how multidisciplinary collaboration between mental health professionals and surgeons influences patient selection and satisfaction could inform clinical best practices.

Additionally, future research should address gaps related to cultural and sociodemographic factors, as perceptions of beauty, self-image, and satisfaction can vary significantly across populations. Diverse and inclusive sampling is essential to understanding how intersecting variables (age, gender identity, cultural background, and media influence) interact with psychological well-being and surgical expectations. More work is needed to understand the trajectory of satisfaction and psychological adjustment beyond the immediate postoperative period. Long-term follow-up studies evaluating outcomes over several years would clarify whether aesthetic procedures contribute to sustained mental health improvements or whether initial satisfaction is short-lived, particularly among patients with underlying psychological conditions.

## 5. Conclusions

This review highlights an association between preoperative psychological disorders (BDD, depression, and anxiety) and reduced postoperative satisfaction among aesthetic surgery patients. The findings demonstrate that individuals with moderate to severe psychological symptoms are at increased risk of dissatisfaction, regardless of surgical success. This dissatisfaction often stems from persistent negative self-image or unmet emotional expectations, which surgery alone cannot resolve. Overall, the findings support integrating structured psychological screening protocols and triage algorithms, such as the SAGA framework, into routine preoperative assessment in aesthetic surgery. Such standardized approaches may facilitate early identification of at-risk patients, guide referral to mental health professionals when appropriate, and improve ethical decision-making and postoperative satisfaction.

## Figures and Tables

**Table 1:** Overview of included studies.

Study	Country	Study Design	Number of Participants	Psychological Assessment Methods	Procedure Type	Primary Outcomes Measured	Satisfaction Measure	Effect Size		Key Findings
Hessler 2010 [26]	Germany	Prospective cohort study	51	BBDQ, structured clinical interview	Facial plastic surgery	Postoperative patient satisfaction following facial plastic	Facial Plastic Surgery Outcomes Questionnaire (FPSOQ) 10-point Likert scale for global satisfaction likelihood of recommending the surgeon	*p* = 0.05		Positive correlation between being treated for depression and satisfaction
Honigman 2011 [25]	Australia	Prospective cohort study	84	GHQ-30, HADS, RSE, DCQ, MBSRQ, PreFACE	Facial cosmetic surgery and cosmetic dentistry	Patient satisfaction	Postoperative questionnaire			Negative correlation between depression and anxiety and satisfaction
Von Soest 2011 [23]	Norway	Longitudinal cohort study	130	Appearance satisfaction	Various cosmetic surgeries	Appearance satisfaction, self-esteem	Postoperative questionnaire	N/A		Negative correlation between preoperative psychological problems (Including depression) and satisfaction
Margraf 2013 [24]	Germany	Prospective observational study	544	German Berner Fragebogen zum Wohlbefinden, German version of the Rosenberg Self-Esteem, EuroQol 5D questionnaire	Various aesthetic surgeries	Psychological outcomes, quality of life	Goal Attainment Scale (self-reported)	N/A		No significant relationship identified
Picavet 2013 [18]	Belgium	Prospective observational study	166	Modified Yale-Brown Obsessive Compulsive Scale, BDDQ, BDD-YBOCS	Rhinoplasty	Postoperative satisfaction, quality of life	Visual analog scale, Rhinoplasty Outcome Evaluation	rho = −0.43 to −0.48, *p* < 0.001		Negative correlation between BDD symptoms (which can include depressive and anxiety symptoms) and satisfaction
Felix 2014 [19]	Brazil	Prospective cohort study	31	BDDE	Rhinoplasty	BDD severity, patient satisfaction	Single self-reported satisfaction item	N/A		No specific findings related to depression or anxiety
Constantian 2014 [21]	USA	Retrospective Cross-sectional study	100	Structured surgeon interview assessing depression, demanding behavior, trauma history, and motivational factors		Secondary rhinoplasty	Patient-defined surgical success	N/A	N/A	Negative correlation between depression and satisfaction
Gabrielyan 2015 [30]	Russia	Cross-sectional study	80	SIBID, SF-36, BIQLI		Various cosmetic surgeries	Body Image Dysphoria, Quality of Life	SF-36 scale	r = −0.7, *p* < 0.05	Negative correlation between BID and Mental Health
de Brito 2016 [28]	Brazil	Prospective cross-sectional study	90	BDDE, BSQ		Abdominoplasty	BDD symptoms, body weight concerns	N/A	N/A	No significant relationship identified
Mendes 2023 [14]	Brazil	Retrospective Observational study	524	Structured clinical interview and Self-report screening tools		Various aesthetic surgeries	Prevalence of psychological disorders	N/A	N/A	No significant relationship identified
Losorelli 2024 [20]	Italy	Retrospective cross-sectional study	115	BDDQ	Rhinoplasty	BDD symptoms, Aesthetic satisfaction	VAS-C (Visual Analog Scale—Cosmesis) SCHNOS-C (Cosmesis domain)	*p* < 0.002		Negative correlation between positive BDD screening and aesthetic satisfaction
Bresnick 2024 [27]	USA	Cohort cross-sectional study	240	DSM-IV/DSM-5 diagnosis of anxiety and/or depression	Breast implant surgery	BII	Postoperative questionnaire	N/A		No significant relationship identified
Wei 2024 [29]	China	Prospective observational study	488	BDDQ	Rhinoplasty, aging face procedures, injectables	BDD screening outcome	Indirect only (SCHNOS-C, Question 5)	N/A		No significant relationship identified

N/A—not applicable; BII—Self-reported breast implant illness; General Health Questionnaire-30 (GHQ-30); Hospital Anxiety and Depression Scale (HADS), Rosenberg Self-Esteem Scale (RSE); Dysmorphic Concerns Questionnaire (DCQ); Multidimensional Body-Self Relations Questionnaire (MBSRQ); PreFACE; Body Dysmorphic Disorder version of the Yale-Brown Obsessive-Compulsive Scale (BDD-YBOCS); Situational Inventory of Body Image Dysphoria (SIBID); Body Image Quality of Life Inventory (BIQLI); Body Dysmorphic Disorder Examination (BDDE); Body Shape Questionnaire (BSQ).

## Supplementary Materials

The following supporting information can be downloaded at: https://www.mdpi.com/article/10.3390/medicina62020389/s1. Table S1: Excluded Studies and Reason for Exclusion; Table S2: QUIPS for Risk of Bias Assessment; Table S3: Risk of Bias Ratings, File S1: PRISMA 2020 Checklist. Ref. [53] is cited in the Supplementary Materials.

## Abbreviations

The following abbreviations are used in this manuscript:

PRISMA	Preferred Reporting Items for Systematic Reviews and Meta-Analyses
PROSPERO	International prospective register of systematic reviews
BDD	Body Dysmorphic Disorder
HADS	Hospital Anxiety and Depression Scale
SIBID	Situational Inventory of Body Image Dysphoria
BDI	Beck Depression Inventory
RSE	Rosenberg Self-Esteem Scale
BSQ	Body Shape Questionnaire
SF-36	Short Form Health Survey (36 items)
GHQ-30	General Health Questionnaire-30
DCQ	Dysmorphic Concerns Questionnaire
MBSRQ	Multidimensional Body-Self Relations Questionnaire
BDD-YBOCS	Body Dysmorphic Disorder version of the Yale-Brown Obsessive-Compulsive Scale
BIQLI	Body Image Quality of Life Inventory
BDDE	Body Dysmorphic Disorder Examination
BII	Breast Implant Illness
