# Supplementary material for: Psychological Disorder and Patient Satisfaction in Aesthetic Surgery—A Systematic Review
Source: Medicina (Kaunas). 2026 Feb 16;62(2):389. doi: 10.3390/medicina62020389 (PMC12942296; doi:10.3390/medicina62020389)
Supplement: Supplementary file 1 [file medicina-62-00389-s001.zip › medicina-4063997-supplementary.pdf]

Supplementary Material

Table S1. Excluded Studies and Reason for Exclusion.

Study	Title	Reason for exclusion
**Jafferany M., 2020**	Psychological aspects of aesthetic and cosmetic surgery: Clinical and therapeutic implications	Insufficient data on prognostic factors
**Hohenberger R., 2021**	Validating the Body Dysmorphic Disorder Questionnaire-Aesthetic Surgery in a German rhinoplasty population	Insufficient data
**Wang Q., 2016**	Avoiding Psychological Pitfalls in Aesthetic Medical Procedures	Wrong population
**Kam O., 2022**	The Psychological Benefits of Cosmetic Surgery	Wrong outcome
**Wever C.C.C., 2020**	Psychiatric Disorders in Facial Plastic Surgery	Wrong outcome
**Sun M., 2021**	How We Do It: Body Dysmorphic Disorder for the Cosmetic Dermatologist	Prognostic factor not included in the outcome study
**Mowlavi A., 2004**	Aspects of plastic surgery. Social and psychological sequelae	Wrong outcome
**Bellino S., 2006**	Dysmorphic concern symptoms and personality disorders: a clinical investigation in patients seeking cosmetic surgery	Insufficient data on prognostic factors
**Veale D., 2014**	Psychological characteristics and motivation of women seeking labiaplasty.	Insufficient data on prognostic factors
**Harth W., 2007**	Psychosomatic disturbances and cosmetic surgery.	Wrong outcome

Table S2. QUIPS for Risk of Bias Assessment

**Domain**	Criteria	Rating of bias risk
**1. Study enrolment**	This domain evaluated whether the study clearly described its sample population, particularly focusing on patients undergoing elective aesthetic surgery with pre-existing psychological conditions such as body dysmorphic disorder (BDD), depression, or anxiety. To be rated at low risk, studies needed to report well-defined eligibility criteria, recruitment settings (e.g., cosmetic clinics, psychiatric screening), and confirm that no major subgroups (e.g., patients with untreated psychiatric illness) were systematically excluded without justification.	a. Low Risk: All criteria are met, suggesting minimal bias<br>in analysis and reporting.<br>b. Moderate Risk: Some criteria are unclear or problematic.<br>c. High Risk: Multiple criteria are problematic, indicating<br>potential bias in analysis and reporting.
**2. Study attrition**	In studies involving aesthetic surgery patients, it is important to monitor whether individuals who dropped out or failed to complete postoperative satisfaction assessments were systematically different from those who remained (e.g., in psychological profile or dissatisfaction with results). Studies were assessed on whether they transparently reported dropout rates, reasons for attrition, and whether follow-up completion was consistent across groups.	a. Low Risk: All criteria are met, suggesting minimal bias<br>in analysis and reporting.<br>b. Moderate Risk: Some criteria are unclear or problematic.<br>c. High Risk: Multiple criteria are problematic, indicating<br>potential bias in analysis and reporting.
**3. Measurement of Psychological Variables**	This domain assessed the validity, consistency, and application of tools used to measure psychological conditions. Instruments like the BDDQ, HADS, BDI, and SIBID had to be applied uniformly and interpreted accurately. We also considered whether the psychological assessments were conducted by qualified professionals (e.g., psychiatrists or psychologists), and whether appropriate diagnostic thresholds or criteria (e.g., DSM-based) were used. Missing or poorly described assessment procedures increased the risk of bias.	a. Low Risk: All criteria are met, suggesting minimal bias<br>in analysis and reporting.<br>b. Moderate Risk: Some criteria are unclear or problematic.<br>c. High Risk: Multiple criteria are problematic, indicating<br>potential bias in analysis and reporting.

**4. Outcome measurement (Patient Satisfaction)**	This criterion evaluated how well postoperative satisfaction was measured regarding psychological status. Studies needed to use validated tools (e.g., BODY-Q, Visual Analog Scale) at consistent time points. We examined whether the measurement scales were appropriate for the type of surgery and whether all participants were assessed under similar conditions. Variability in tools or vague outcome descriptions was flagged as moderate or high risk.	a. Low Risk: All criteria are met, suggesting minimal bias in analysis and reporting.<br>b. Moderate Risk: Some criteria are unclear or problematic.<br>c. High Risk: Multiple criteria are problematic, indicating potential bias in analysis and reporting.
**5. Adjustment for Confounders**	We examined whether studies accounted for relevant confounding factors, such as severity of psychological disorders, type of aesthetic surgery, patient expectations, age, or previous surgical history. Studies that used multivariate models or stratified analyses to isolate the influence of psychological conditions on satisfaction were considered at low risk. Failure to consider major confounders indicated higher bias risk.	a. Low Risk: All criteria are met, suggesting minimal bias in analysis and reporting.<br>b. Moderate Risk: Some criteria are unclear or problematic.<br>c. High Risk: Multiple criteria are problematic, indicating potential bias in analysis and reporting.
**6. Statistical Analysis and Reporting**	This domain assessed whether the statistical methods used were suitable for studying the link between psychological status and satisfaction. Low-risk studies reported effect sizes, *p*-values, and confidence intervals, and avoided selective reporting (e.g., omitting non-significant results). Analyses had to match the study design and include relevant subgroup or regression analyses if multiple psychological factors were considered. Studies that lacked transparency or reported only favorable results were rated at higher risk.	a. Low Risk: All criteria are met, suggesting minimal bias in analysis and reporting.<br>b. Moderate Risk: Some criteria are unclear or problematic.<br>c. High Risk: Multiple criteria are problematic, indicating potential bias in analysis and reporting.

Table S3. Risk of Bias Ratings

**Study**	D1. Study participation	D2. Study attrition	D3. Measurement of Psychological Variables	D4. Outcome measurement (Patient Satisfaction)	D5. Adjustment for Confounders	D6. Statistical Analysis and Reporting
**Hessler 2010**	Low	Low	Low	Low	Moderate	Low
**Honigman 2011**	Moderate	High	High	Moderate	High	Moderate
**Von Soest 2011**	Low	Low	Low	Low	Low	Low
**Margraf 2013**	Low	Moderate	Low	Low	Moderate	Low
**Picavet 2013**	Moderate	Moderate	Moderate	Moderate	High	Moderate
**Felix 2014**	Low	Low	Low	Low	Moderate	Low
**Constantian 2014**	Low	Moderate	High	Moderate	High	Moderate
**Gabrielyan 2015**	Low	Moderate	Moderate	Low	Moderate	Moderate
**de Brito 2016**	Low	Low	Low	Low	Moderate	Low
**Mendes 2023**	Low	Low	Low	Low	Low	Low
**Losorelli 2024**	Low	Low	Low	Low	Moderate	Moderate
**Bresnick 2024**	Moderate	Moderate	Moderate	Low	Moderate	Low
**Wei 2024**	Moderate	High	Moderate	Moderate	Moderate	High
