# Supplementary material for: Psychological Disorder and Patient Satisfaction in Aesthetic Surgery—A Systematic Review
Source: Medicina (Kaunas). 2026 Feb 16;62(2):389. doi: 10.3390/medicina62020389 (PMC12942296; doi:10.3390/medicina62020389)
Supplement: Supplementary file 1 [file medicina-62-00389-s001.zip › medicina-4063997-PRISMA 2020 Checklist.pdf]

PRISMA 2020 Checklist

Section and Topic	Item #	Checklist item	Location where item is reported
**TITLE**			
Title	1	Psychological Disorder and Patient Satisfaction in Aesthetic Surgery – A Systematic Review	Lines 2-3
**ABSTRACT**			
Abstract	2	**Background**: This systematic review investigates the relationship between preoperative psychological disorders and postoperative satisfaction among patients undergoing aesthetic surgery. While aesthetic procedures can enhance self-image, growing evidence indicates that underlying mental health conditions, particularly BDD, depression, and anxiety, may compromise surgical outcomes. **Methods**: Following PRISMA guidelines, thirteen peer-reviewed studies were analyzed to evaluate how these psychological factors influence satisfaction and to assess the adequacy of preoperative psychological screening. **Results**: The findings reveal a consistent pattern: patients exhibiting moderate to severe psychological symptoms before surgery are more likely to report dissatisfaction, regardless of the technical success of the procedure. Conversely, favorable outcomes are observed when psychological conditions are mild or effectively managed through structured assessments. The review also highlights considerable methodological variability across studies, especially in psychological screening tools and follow-up duration, limiting meta-analytic integration. Despite these differences, the evidence underscores the need for standardized, validated psychological evaluation protocols in aesthetic surgery. Incorporating mental health screening into routine surgical planning can enhance ethical practice, reduce dissatisfaction, and improve long-term patient outcomes. **Conclusions**: These findings advocate for a multidisciplinary approach that includes psychological assessment as an essential component of patient care in aesthetic medicine.	Lines 23-41
**INTRODUCTION**			
Rationale	3	The rationale behind this systematic review stems from growing evidence that psychological disorders—particularly Body Dysmorphic Disorder (BDD), depression, and anxiety—may significantly influence postoperative satisfaction in aesthetic surgery patients. While cosmetic procedures are often pursued to enhance self-image and emotional well-being, unrecognized or untreated mental health conditions can undermine surgical outcomes, leading to persistent dissatisfaction despite technically successful interventions. The review identifies a crucial gap in the literature, noting the inconsistent use of psychological screening methods and the fragmented nature of existing studies. As a result, this research aims to synthesize current findings, evaluate the impact of preoperative psychological disorders on patient satisfaction, and assess the adequacy of mental health evaluations in aesthetic surgical practice. Ultimately, it advocates for the integration of standardized psychological assessments into preoperative planning to enhance patient outcomes and ethical standards in aesthetic medicine.	Lines 45-88
Objectives	4	This review aims to evaluate the impact of preoperative psychological disorders on postoperative satisfaction among aesthetic surgery patients and to assess the adequacy of current psychological assessment practices used in this context. Specifically, the review seeks to (1) identify and classify the most frequently reported psychological conditions among cosmetic surgery candidates; (2) examine how these conditions influence postoperative satisfaction and perceived surgical outcomes; and (3) assess the quality and consistency of preoperative psychological screening methods used in aesthetic surgical practice. By addressing these objectives, the study aims to better understand the mental health risks and considerations relevant to patients and practitioners.	Lines 89-97
**METHODS**			
Eligibility criteria	5	**Inclusion criteria:**<br>Adults (≥18 years) undergoing elective aesthetic surgery<br>Diagnosed preoperative psychological disorders (e.g., BDD, depression, anxiety)<br>Documented psychological assessment before surgery<br>Original, peer-reviewed research using observational study designs<br>Reported patient satisfaction outcomes<br>Minimum sample size of 10 participants<br>English-language publications (2011–2024)<br>**Exclusion criteria:**	Lines 121-137

PRISMA 2020 Checklist

Section and Topic	Item #	Checklist item	Location where item is reported
		Non-surgical or medically indicated procedures<br>No psychological assessment<br>No satisfaction data<br>Incomplete methodological details	
Information sources	6	**Databases searched:**<br>PubMed, Cochrane Library, Google Scholar<br>**Search limits:**<br>Studies published January 2011 – December 2024<br>Only peer-reviewed, English-language, human studies<br>Additional sources:<br>Reference lists of included studies<br>Date of last search:<br>December 2024	Lines 105-116
Search strategy	7	**Keywords and combinations used:**<br>"psychological disorders" AND "aesthetic surgery"<br>"body dysmorphic disorder" OR "BDD" AND "cosmetic surgery"<br>"mental health in plastic surgery"<br>"psychiatric evaluation in cosmetic procedures"<br>**Filters and limits applied:**<br>Publication date: 2011–2024<br>Language: English<br>Study type: Original, peer-reviewed articles involving human participants	Lines 105-116
Selection process	8	Two reviewers independently screened all records at the title, abstract, and full-text levels to determine eligibility. They applied the predefined inclusion and exclusion criteria consistently. Disagreements were resolved through discussion.	Lines 118-130
Data collection process	9	An automation tool (Elicit AI) was used to assist in data extraction, but not in the initial screening phase. No authors were contacted for additional data.	Lines 118-130
Data items	10a	**Outcomes sought:**<br>Primary outcome:<br>Postoperative patient satisfaction<br>Defined as self-reported satisfaction with aesthetic surgical results.<br>Secondary data points (supporting satisfaction):<br>Measurement instruments used (e.g., Visual Analog Scale, BODY-Q, Rhinoplasty Outcome Evaluation)<br>Timing of satisfaction assessments (e.g., short- vs. long-term follow-up)<br>Scoring details (range, interpretation)	Lines 139-147

Section and Topic	Item #	Checklist item	Location where item is reported
		**Data collection approach:**<br>All available satisfaction results were extracted if they fit the outcome domain.<br>When multiple satisfaction measures were reported, validated and surgery-specific tools were prioritized.<br>If results lacked clear definitions or measures, they were noted as "N/A"; no assumptions were made.	
	10b	**Participant characteristics:**<br>Age, gender, sample size, country/setting<br>**Psychological conditions:**<br>Diagnosed preoperative disorders: Body Dysmorphic Disorder (BDD), depression, anxiety<br>**Assessment methods:**<br>Psychological tools used (e.g., BDDQ, HADS, BDI, SIBID, BDDE)<br>Presence or absence of structured clinical interviews<br>**Surgical characteristics:**<br>Type of aesthetic procedure (e.g., rhinoplasty, breast augmentation, facelifts)<br>**Study design details:**<br>Study type (e.g., prospective, cross-sectional), data collection method<br>**Funding sources:**<br>Institutional support (noted where applicable)	Lines 148-166
Study risk of bias assessment	11	**Tool used: QUIPS (Quality In Prognosis Studies)**<br>Domains assessed (6): study participation, attrition, prognostic factor measurement, outcome measurement, confounding control, statistical analysis/reporting<br>**Review process:**<br>Initial classification was done using a large language model (LLM)<br>Then reviewed and corrected by a single human reviewer	Lines 167-176
Effect measures	12	Correlation coefficients (e.g., Pearson's r, Spearman's ρ): to quantify associations between psychological conditions and satisfaction<br>p-values: to indicate statistical significance of the associations<br>Effect sizes (where reported): to interpret the strength of the relationship (e.g., moderate to strong negative correlation)	Lines 178-187
Synthesis methods	13a	The authors tabulated key characteristics of each included study (e.g., psychological disorder assessed, type of aesthetic procedure, study design, and satisfaction measures).<br>Studies were grouped based on their compatibility with the review objectives:<br>• Presence of preoperative psychological assessment<br>• Reporting of postoperative satisfaction outcomes<br>• Use of validated tools (psychological and satisfaction measures)<br>Only studies that met all inclusion criteria and provided sufficient methodological detail were included in the final synthesis (n = 13). Those lacking psychological evaluation, satisfaction data, or clear design descriptions were excluded (as detailed in Table S1).	Lines 178-187

Section and Topic	Item #	Checklist item	Location where item is reported
	13b	No data conversions or imputations were performed. Missing or unclear data (e.g., absent satisfaction scores or unspecified psychological tools) were marked as "N/A". Only explicitly reported, validated outcomes were included in the synthesis.	Lines 178-187
	13c	Results were tabulated in detailed summary tables and visually displayed using a PRISMA flow diagram and figures showing procedure distribution and outcome relationships.	Lines 178-187
	13d	A narrative synthesis was used due to high methodological heterogeneity across studies (e.g., differences in psychological assessments, procedures, and satisfaction tools). Data analysis and visualization were performed using Python 3.11	Lines 178-187
	13e	Not applicable	-
	13f	No sensitivity analyses were conducted.	-
Reporting bias assessment	14	No formal methods (e.g., funnel plots or statistical tests) were used to assess risk of bias due to missing results or reporting biases.	-
Certainty assessment	15	No formal methods (e.g., GRADE) were used to assess the certainty or confidence in the body of evidence.	-
**RESULTS**			
Study selection	16a	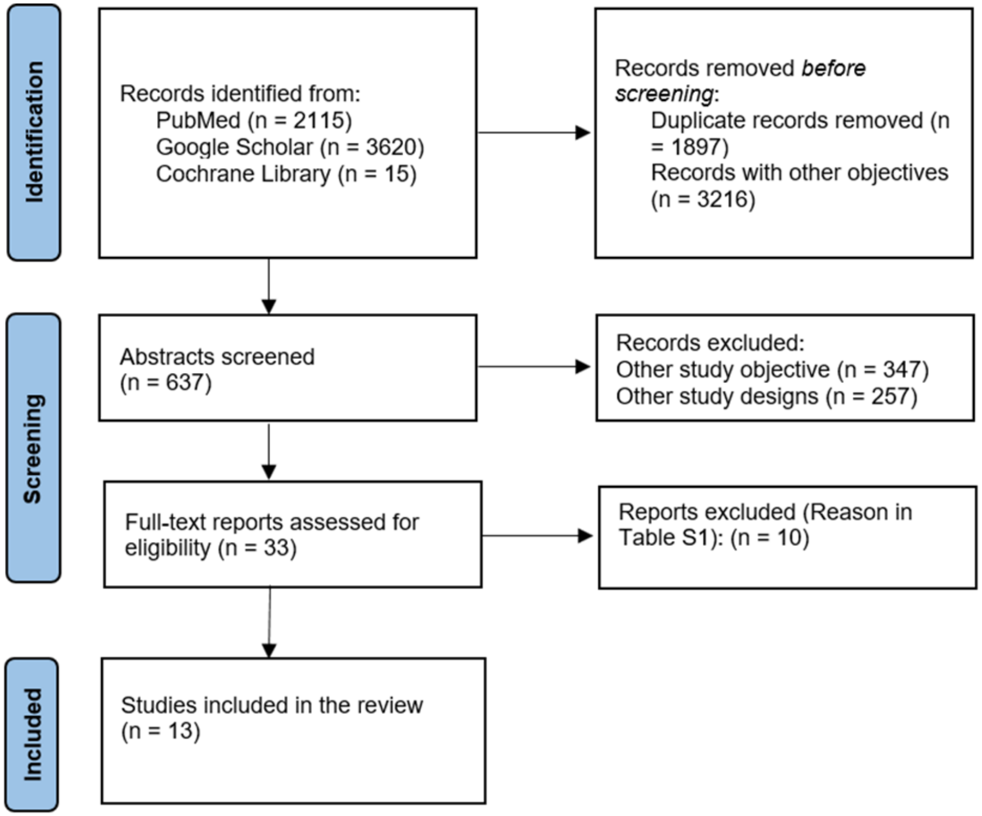	Line 131

Section and Topic	Item #	Checklist item	Location where item is reported
	16b	**Study** / **Title** / **Reason for exclusion**<br>**Jafferany M., 2020** — Psychological aspects of aesthetic and cosmetic surgery: Clinical and therapeutic implications — Insufficient data on prognostic factors<br>**Hohenberger R., 2021** — Validating the Body Dysmorphic Disorder Questionnaire-Aesthetic Surgery in a German rhinoplasty population — Insufficient data<br>**Wang Q., 2016** — Avoiding Psychological Pitfalls in Aesthetic Medical Procedures — Wrong population<br>**Kam O., 2022** — The Psychological Benefits of Cosmetic Surgery — Wrong outcome<br>**Wever C.C.C., 2020** — Psychiatric Disorders in Facial Plastic Surgery — Wrong outcome<br>**Sun M., 2021** — How We Do It: Body Dysmorphic Disorder for the Cosmetic Dermatologist — Prognostic factor not included in the outcome study<br>**Mowlavi A., 2004** — Aspects of plastic surgery. Social and psychological sequelae — Wrong outcome<br>**Bellino S., 2006** — Dysmorphic concern symptoms and personality disorders: a clinical investigation in patients seeking cosmetic surgery — Insufficient data on prognostic factors<br>**Veale D., 2014** — Psychological characteristics and motivation of women seeking labiaplasty. — Insufficient data on prognostic factors<br>**Harth W., 2007** — Psychosomatic disturbances and cosmetic surgery. — Wrong outcome	Supplementary Material Table S1.
Study characteristics	17	14. Picavet, V.A.; Gabriëls, L.; Grietens, J.; Jorissen, M.; Prokopakis, E.P.; Hellings, P.W. Preoperative Symptoms of Body Dysmorphic Disorder Determine Postoperative Satisfaction and Quality of Life in Aesthetic Rhinoplasty. Plastic and Reconstructive Surgery 2013, 131, 861–868, doi:10.1097/prs.0b013e3182818f02.<br>Showed that preoperative body dysmorphic symptoms strongly predict lower satisfaction and poorer quality of life after rhinoplasty<br>15. Felix, G.A.A.; de Brito, M.J.A.; Nahas, F.X.; Tavares, H.; Cordás, T.A.; Dini, G.M.; Ferreira, L.M. Patients with Mild to Moderate Body Dysmorphic Disorder May Benefit from Rhinoplasty. Journal of Plastic, Reconstructive & Aesthetic Surgery 2014, 67, 646–654, doi:10.1016/j.bjps.2014.01.002.<br>Demonstrated that patients with mild to moderate body dysmorphic disorder can benefit from rhinoplasty, with high satisfaction and symptom remission rates.<br>16. Losorelli, S.; Kimura, K.S.; Wei, E.X.; Abdelhamid, A.S.; El Abany, A.; Green, A.; Karki, S.; Stephanian, B.A.; Kandathil, C.K.; Most, S.P. Rhinoplasty Outcomes in Patients With Symptoms of Body Dysmorphia. Aesthetic Surgery Journal 2024, 44, 797–804, doi:10.1093/asj/sjae045.<br>Investigated how rhinoplasty impacts patients with body dysmorphic symptoms, showing high rates of postoperative symptom resolution and improved satisfaction<br>17. Constantian, M.B.; Lin, C.P. Why Some Patients Are Unhappy. Plastic and Reconstructive Surgery 2014, 134, 836–851, doi:10.1097/prs.0000000000000552.<br>Found that trauma history and normal nasal shape correlate with dissatisfaction and repeated rhinoplasty, linking body dysmorphia to past	Lines 234-248

Section and Topic	Item #	Checklist item	Location where item is reported
		abuse<br>19. von Soest, T.; Kvalem, I.L.; Skolleborg, K.C.; Roald, H.E. Psychosocial Changes after Cosmetic Surgery. Plastic and Reconstructive Surgery 2011, 128, 765–772, doi:10.1097/prs.0b013e31822213f0.<br>Demonstrated long-term (5-year) improvements in appearance satisfaction after cosmetic surgery, but minimal changes in self-esteem or mental health, especially among psychologically vulnerable patients<br>20. Margraf, J.; Meyer, A.H.; Lavallee, K.L. Well-Being From the Knife? Psychological Effects of Aesthetic Surgery. Clinical Psychological Science 2013, 1, 239–252, doi:10.1177/2167702612471660.<br>Found sustained psychological benefits (e.g., reduced anxiety and depression) in aesthetic surgery patients compared to controls over a 12-month period<br>21. Honigman, R.J.; Jackson, A.C.; Dowling, N.A. The PreFACE. Annals of Plastic Surgery 2011, 66, 16–23, doi:10.1097/sap.0b013e3181d50e54.<br>Developed the PreFACE screening tool to identify psychosocial risk factors predicting dissatisfaction after facial cosmetic surgery<br>22. Hessler, J.L.; Moyer, C.A.; Kim, J.C.; Baker, S.R.; Moyer, J.S. Predictors of Satisfaction With Facial Plastic Surgery. Arch Facial Plast Surg 2010, 12, doi:10.1001/archfacial.2009.69.<br>Identified age and current depression treatment as predictors of higher satisfaction after facial plastic surgery<br>23. Bresnick, S.; Lagman, C.; Morris, S.; Bresnick, S.; Robbins, M. Correlation Between Medically Diagnosed Anxiety and Depression Disorder and Self-Reported Breast Implant Illness. Aesthetic Surgery Journal 2024, 44, 1118–1126, doi:10.1093/asj/sjae089.<br>24. Marron Mendes, V.; Diluiso, G.; Jidjouc Kamdem, C.; Goulliart, S.; Schettino, M.; Dziubek, M.; Di Fiore, C.; Ortiz Carrillo, S.; Delhaye, M. Prevalence of Psychiatric Disorders in Aesthetic Surgery. Ann Plast Surg 2023, 91, 413–421, doi:10.1097/sap.0000000000003682.<br>Reported a 32.25% prevalence of psychiatric disorders among aesthetic surgery seekers, recommending multidisciplinary management<br>25. de Brito, M.J.A.; Nahas, F.X.; Cordás, T.A.; Gama, M.G.; Sucupira, E.R.; Ramos, T.D.; Felix, G. de A.A.; Ferreira, L.M. Prevalence of Body Dysmorphic Disorder Symptoms and Body Weight Concerns in Patients Seeking Abdominoplasty. Aesthet Surg J 2016, 36, 324–332, doi:10.1093/asj/sjv213.<br>Reported a 57% prevalence of body dysmorphic symptoms among abdominoplasty candidates, with severity tied to body image and weight concerns<br>26. Wei, Z. Dependent Personality Disorder and Its Gender Differences. LNEP 2023, 2, 349–354, doi:10.54254/2753-7048/2/2022487.<br>Identified a 41% prevalence of positive body dysmorphic disorder (BDD) screening in facial cosmetic surgery patients, especially among younger individuals and those with psychiatric histories<br>27. Gabrielyan, A.; Sukiasyan, S. Body Image Dysphoria and Quality of Life among Women Who Apply for Cosmetic Surgery. 2015.<br>Showed that while cosmetic surgery improves body image dysphoria and some quality of life aspects, issues with body acceptance often persist	
Risk of bias in studies	18	Picavet et al. – Low risk of bias: This is a well-designed prospective study with an adequate sample size (n=166), use of validated instruments (e.g., ROE, DAS-59, Sheehan Disability Scale), and follow-up at 3 and 12 months. Preoperative BDD symptoms were systematically assessed and statistically correlated with postoperative satisfaction and quality of life, supporting the study's internal validity.<br>Losorelli et al. – Moderate risk of bias: lacks a control group and relies on self-reported symptoms post-rhinoplasty, which can be subjective.<br>Constantian et al. – High risk of bias: retrospective design with subjective assessments and unclear diagnostic criteria for body dysmorphic disorder (BDD).	Supplementary Material Table S2.

Section and Topic	Item #	Checklist item	Location where item is reported
		De Brito et al. – Moderate to high risk of bias: cross-sectional design with limited psychological diagnostic rigor; relies on self-reporting.<br>Felix et al. – Moderate risk of bias: prospective design but lacks control group and long-term follow-up; sample size may be limited.<br>Gabrielyan et al. – Moderate risk of bias: small sample and subjective measures of body image; lacks randomization or comparison group.<br>Hessler et al. – Low to moderate risk of bias: prospective design with pre- and post-surgery assessments, but small sample size and limited generalizability.<br>Honigman et al. – Moderate risk of bias: development of a screening tool without full external validation; may reflect researcher assumptions.<br>Margraf et al. – Low risk of bias: controlled prospective study with matched samples and standardized psychological measures.<br>Mendes et al. – Moderate risk of bias: retrospective, observational study relying heavily on recorded clinical histories, which may omit psychiatric data.<br>Picavet et al. – Low risk of bias: well-designed prospective study with validated instruments and long-term follow-up.<br>Von Soest et al. – Low to moderate risk of bias: strong 5-year follow-up design, but potential selection bias due to attrition and lack of randomization.<br>Wei et al. – Low to moderate risk of bias: robust screening of a large clinical sample, though limited by being cross-sectional and clinic-based.	
Results of individual studies	19	(see table below)	Lines 234-248

Study	Groups	Summary Statistics	Effect Estimate (and Precision)
**Losorelli et al.**	Post-rhinoplasty BDD resolved vs not resolved	92.8% had improvement; 75.7% symptom-free	Descriptive only; no effect size reported
**Constantian et al.**	BDD-like symptoms with trauma vs without	33% had abuse histories; many had normal nasal anatomy	Observational; no statistical estimates
**De Brito et al.**	BDD symptoms vs none	57% prevalence; higher dissatisfaction & anxiety	Descriptive; cross-sectional
**Felix et al.**	Mild/moderate BDD pre-op vs post-op	81% post-op satisfaction; 60% BDD remission	Descriptive; no CI reported
**Gabrielyan et al.**	Pre/post cosmetic surgery	Improved dysphoria & appearance anxiety	No confidence intervals provided
**Hessler et al.**	Satisfied vs not after surgery	Satisfaction linked to age and depression treatment	Qualitative associations only
**Honigman et al.**	Risk factor presence vs none	Tool predicted dissatisfaction	Validation stage; no effect sizes
**Margraf et al.**	Surgery vs no surgery vs dental vs control	Improved well-being in surgery group	$p < 0.01$; no CI reported
**Mendes et al.**	Psychiatric history vs none	32.25% had psychiatric disorders; differences in addictions	Significant differences; $p < 0.05$

Section and Topic	Item #	Checklist item	Location where item is reported

**Picavet et al.**	Low vs high BDD symptoms pre-op	Higher BDD = lower satisfaction and QoL	rho ≈ -0.40 to -0.48, p < 0.001
**Von Soest et al.**	Pre vs 5-year post-surgery	↑ Appearance satisfaction (d=0.41), ↓ dissatisfaction (d=0.67)	Cohen's d: 0.18–0.67, p < 0.01
**Wei et al.**	Positive BDD screen vs negative	41% screened positive; linked to age, revision, psych history	Significant predictors; p < 0.01

Section and Topic	Item #	Checklist item	Location where item is reported
Results of syntheses	20a	**Study Characteristics (n = 13):**<br>Designs: Predominantly prospective or observational cohort studies; some cross-sectional<br>Sample sizes: Ranged from 31 to 544 participants<br>Procedures studied: Mostly rhinoplasty, breast augmentation, facial cosmetic surgery, and abdominoplasty<br>Psychological assessments: Varied – included validated tools like BDDQ, BDI, HADS, BDDE, SIBID; some studies lacked formal tools<br>Satisfaction measurement: Visual Analog Scales, surgery-specific tools (e.g., Rhinoplasty Outcome Evaluation), and general QoL scales (e.g., SF-36)<br>**Risk of Bias Summary:**<br>Low risk of bias:<br>Studies like Hessler (2010), Von Soest (2011), Felix (2014), de Brito (2016), Losorelli (2024) – used validated psychological tools, had clear designs, and consistent outcome reporting.<br>Moderate risk of bias:<br>Picavet (2013), Gabrielyan (2015) – lacked full adjustment for confounders or detailed description of methods.<br>High risk of bias:<br>Honigman (2011), Constantian (2014), Wei (2024) – used unclear or unvalidated tools, had insufficient confounder control, and/or high attrition risk.	Lines 203-225 and Lines 249-276
	20b	No meta-analysis was conducted in this review due to substantial heterogeneity across studies (in designs, psychological measures, and satisfaction outcomes).<br>**Body Dysmorphic Disorder (BDD):**<br>Picavet (2013):<br>ρ = −0.43 to −0.48, p < 0.001<br>Direction: Higher BDD symptoms → lower satisfaction<br>Losorelli (2024):<br>p < 0.002<br>Direction: Positive BDD screening → lower aesthetic satisfaction<br>Gabrielyan (2015):<br>r = −0.7, p < 0.5<br>Direction: Body image dysphoria → poorer mental health QoL	Lines 233-248

Section and Topic	Item #	Checklist item	Location where item is reported
		**Depression and Anxiety:**<br>Honigman (2011):<br>Negative correlation between depression/anxiety and satisfaction (exact values not reported)<br>Direction: More symptoms → lower satisfaction<br>Constantian (2014):<br>Directional result only: Depression is linked to dissatisfaction<br>Von Soest (2011):<br>Negative association between psychological problems (including depression) and appearance satisfaction<br>Direction: Higher symptoms → lower satisfaction<br>**Non-significant findings:**<br>Felix (2014), de Brito (2016), Mendes (2023), Wei (2024), Bresnick (2024):<br>Reported no statistically significant associations between psychological conditions and satisfaction	
	20c	The review did not perform formal statistical investigations (e.g. subgroup analyses, meta-regression) to explore causes of heterogeneity.	-
	20d	No sensitivity analyses were conducted in this review.	-
Reporting biases	21	The review did not conduct formal assessments of risk of bias due to missing results.	-
Certainty of evidence	22	The review did not use a formal framework (such as GRADE) to assess the certainty or confidence in the body of evidence for any outcome.	-
**DISCUSSION**			
Discussion	23a	The results of this review reinforce and expand upon prior evidence showing that preoperative psychological disorders—especially Body Dysmorphic Disorder (BDD), depression, and anxiety—are significantly associated with reduced postoperative satisfaction in aesthetic surgery. This aligns with earlier findings from Honigman et al., Mulkens et al., and Jafferany et al., who all emphasized that unaddressed psychological distress can undermine the perceived benefits of surgery, regardless of its technical success.<br>The review makes a unique contribution by showing that patients with mild or well-managed symptoms, particularly those who underwent structured psychological screening, often experience better satisfaction outcomes. This supports the growing consensus that mental health screening should be integrated into surgical planning to optimize patient well-being and reduce dissatisfaction.<br>It also contrasts with broader quality-of-life research (e.g., Dreher et al.), which has generally found positive psychosocial outcomes from cosmetic surgery, but without explicitly accounting for psychological comorbidity. This contrast underscores the importance of stratifying patients by psychological risk when evaluating outcomes.<br>In summary, this review confirms that psychological readiness is a key determinant of postoperative satisfaction and underscores the ethical and clinical importance of preoperative mental health evaluation in aesthetic practice.	Lines 321-337
	23b	The evidence included in the review was limited by high methodological heterogeneity, particularly in the use of psychological assessment tools, satisfaction measures, and follow-up timing. Several studies had small sample sizes, lacked validated instruments, or failed to control for confounding factors, thereby reducing the reliability and comparability of their findings.	Lines 484-497
	23c	The review process had several limitations: it did not include a formal GRADE assessment for evidence certainty, and no sensitivity analyses or quantitative exploration of heterogeneity were conducted. Additionally, the risk of bias was assessed by a single reviewer after initial AI support; however, full search strategies (e.g., exact strings) were not reported, which limits reproducibility.	Lines 484-497
	23d	Implications for practice:	Lines 498-516

PRISMA 2020 Checklist

Section and Topic	Item #	Checklist item	Location where item is reported
		The findings underscore the importance of integrating standardized psychological screening, utilizing validated tools, into routine preoperative assessments in aesthetic surgery. Mental health evaluation should be seen not as a barrier, but as a way to enhance patient outcomes and satisfaction.<br>Policy implications:<br>Clinical guidelines and institutional protocols should require multidisciplinary collaboration between plastic surgeons and mental health professionals to ensure ethical, patient-centered care, especially for those at psychological risk.<br>Implications for future research:<br>Future studies should employ longitudinal designs, utilize larger and more diverse populations, and employ validated diagnostic tools to understand better how psychological disorders affect satisfaction over time. Research should also explore the effectiveness of preoperative psychological interventions in improving outcomes.	
**OTHER INFORMATION**			
Registration and protocol	24a	PROSPERO ID:	Lines 102-103
	24b	Link prospero	
	24c	No amendments to the registered protocol were reported or described in the review.	-
Support	25	We want to acknowledge the Victor Babes University of Medicine and Pharmacy, Timisoara, Romania, for covering the publication costs for this research paper.	Lines 528-529
Competing interests	26	The review authors declared no competing interests.	Line 534
Availability of data, code, and other materials	27	The review states that supplementary materials are available, including:<br>Table S1: Excluded studies and reasons for exclusion<br>Table S2: QUIPS risk of bias assessment<br>Table S3: Risk of bias ratings	Supplementary Material

From: Page MJ, McKenzie JE, Bossuyt PM, Boutron I, Hoffmann TC, Mulrow CD, et al. The PRISMA 2020 statement: an updated guideline for reporting systematic reviews. BMJ 2021;372:n71. doi: 10.1136/bmj.n71. This work is licensed under CC BY 4.0. To view a copy of this license, visit https://creativecommons.org/licenses/by/4.0/
